# Knowledge and information sources of potential drug–drug interactions of healthcare professionals among Buraydah Hospitals

**DOI:** 10.1186/s40545-023-00642-0

**Published:** 2023-10-31

**Authors:** Salwa Selim Ibrahim Abougalambou, Tief Naif Alenezi

**Affiliations:** 1 Discipline of Pharmacy Practice, Pharmacy College, Ahram Canadian University, 6th of October City, Giza, Egypt; 2 Clinical Pharmacist, AL Habib Hospital, Sulaiman Al habib, Buraydah, Saudi Arabia

**Keywords:** Healthcare professionals, Drug–drug interactions, Knowledge, Sources of information

## Abstract

**Background and objective:**

Drug–drug interactions (DDI) are known to increase the risk of morbidity and mortality, and adversely affect the patient's quality of life. The study was to assess healthcare professional's (HCP) knowledge of DDIs in general hospitals of Buraydah.

**Methods:**

A cross-sectional survey using convenience sampling methods was conducted among 135 healthcare professionals in general hospitals of Buraydah between November and December 2016. The study was carried out after approval and permission from the Regional Research Ethics Committee (November 2016). Respondents were asked to classify 15 drug pairs as 'contraindicated', 'could be used with monitoring', or 'no interaction'. A response option of 'not sure' was also provided. Data were collected using a self-administered questionnaire. The descriptive analysis was done using frequency distribution and percentage for demographic data and other responses to questions. Data were collected, tabulated, and analyzed using Statistical Package for Social Sciences (SPSS) software (version 23). Logistic regression analysis was used to assess the independent variables that affect the HCP knowledge, the significant levels were set at *p*-value < 0.05.

**Results:**

A total of 135 healthcare professionals were included in the study. The percentage of HCPs who correctly classified the drug pairs ranged from 15 (11.1%) for "Allopurinol + Pyrazinamide" to 90 (66.7%) for "acetaminophen with codeine + amoxicillin". The average number of correctly categorized drug pairs was 5. About one-half of the respondents 73 (54.1%) answered correctly. The level of education was found to be an independent predictor of DDI knowledge. The results from the multivariate analysis indicated that a higher potential DDI knowledge level was associated with pharmacists. Pharmacists had 8.27 times higher DDI knowledge tests than nurses, *P* value = 0.001. Pharmacists 43(31.9%) were the most cited information source.

**Conclusions:**

The present study revealed that health care professional’s DDI knowledge was inadequate. Level of education was significantly associated with healthcare professionals’ DDI knowledge. Pharmacists were the most cited DDI information source. Healthcare professionals should update their DDI knowledge through continuing education and should improve their familiarity with DDI information sources. These updated educations help to provide the appropriate therapeutic outcomes.

## Background

Drugs used to treat patients take a top priority to preserve their health and improve their personal satisfaction and quality of life. With each drug added to the patient’s regimen, this priority can turn into inferiority by causing clinically significant drug–drug interactions (DDIs).

The World Health Organization (WHO) defines adverse drug reaction as any harmful, unintended reaction to medicines that occur at doses normally used for prophylaxis, diagnosis, or treatment [29]. The definition of drug–drug interactions (DDI) is the alteration of one drug’s effect by the presence of another [19]. Drug interactions fall into three different possible mechanisms: pharmacodynamics, pharmacokinetic, or clinical responses of drugs that are administered together, in which the combined effect produced by those drugs is different when either drug is administered alone [18]. The effect of DDI may be considered as desirable or favorable, or on the other hand undesirable or harmful effect [27]. It is important to note that DDI can also cause new side effects that could be life-threatening or even fatal [3].

The prevalence of drug–drug interactions identified in the literature varies due to the different research methods used in each study [26]. For example, DDIs have been studied in community settings, hospital and outpatient settings, and in register-based studies. A prevalence of 26.5–63% was shown in community settings (Teixeira et al. 2012). The prevalence rates also differ among hospital settings, the rates were 57.8% in the Department of Psychiatry [10], whereas in emergency departments they were 0.7% [4]. Lastly, register-based studies reported prevalence rates of 15–26% [22].

DDIs are one important and unrecognized type of medication error that predisposes patients to hospitalization and increases the cost burden on healthcare systems [13]. DDIs are known to increase the risk of morbidity and mortality, and adversely affect the patient's quality of life [23]. Studies indicate that DDIs harm 1.9 to 5 million inpatients per year and cause 2600 to 220,000 emergency department visits per year [16]. It is also stated that since 1995, healthcare costs have doubled due to drug-related problems including DDI [8].

Physicians, nurse practitioners, and pharmacists constitute the group of providers in closest proximity to patients receiving medications. Thus, understanding the degree to which these providers can recognize an interaction and identify a proper management strategy is vital to developing new methods to reduce DDIs [5]. The limited data available suggest that the DDI knowledge of practicing physicians, nurse practitioners, and pharmacists is poor [5, 14].

Nobody can retain all the potential DDIs that have been distinguished to date, and new interfacing drug sets are recognized each month [30]. To adapt to this undertaking drug collaboration compendia as books, diaries, associates, drug specialists, PC or individual advanced colleague programming, or online databases, for example, the US database by Thomson Micromedex™ or the British Greenwood Village or Stockley’s Drug Interactions, Pharmavista®, ABDA-Database and so on are offered to human services suppliers [15].

A study carried out in Saudi Arabia by Al-Arifi et al. [1] to assess HCPs' knowledge on warfarin–drug/herb interactions revealed that HCPs failed to recognize all the potentially harmful warfarin–drug interactions. The same study showed that among anti-inflammatory agents, only aspirin and warfarin were correctly classified by the majority (92.2%) of HCPs. Warfarin and cardiac agents (propranolol) had a moderate recognition rate (53.3%). Another potentiating drug with the effect of warfarin was fluconazole; its low recognition (47.8%) resulted in bleeding if the anticoagulant dosage was not reduced appropriately. In addition, two drugs inhibiting the effect of warfarin including sucralfate and phenytoin had a low recognition rate (27.8% and 31.3%, respectively) of the HCPs. This study also found a low recognition rate for atenolol, ranitidine, and fluoxetine (11.1%, 15.6%, and 4.4%, respectively). These drugs do not affect warfarin action. The study found that pharmacists had slightly higher knowledge than doctors and nurses although the difference was not significant [1].

Ndosi and Newell found that nurses' pharmacological knowledge was quite poor and although a few nurses showed high levels of pharmacological knowledge, the majority had inadequate knowledge (2008). Only 11 (26.1%) nurses scored eight or above and the majority 24 (57.2%) scored below seven, indicating inadequate knowledge. In the same study, the knowledge of drug mechanisms of action and drug interactions was poor [21].

A study carried out by Weideman et al. to assess pharmacists’ recognition of potential drug interactions found that pharmacists cannot identify important drug interactions. The pharmacists were given a set of eight 2-drug profiles, four 4-drug profiles, two 8-drug profiles, and one 16-drug profile. They were able to identify only 66% of the interactions in the 2-drug profiles, 34% of the interactions in the 4-drug profile, 20% of the interactions in the 8-drug profile, and 17% of the interactions in the 16-drug profile. None of them were able to recognize all interactions in the 8- or 16-drug profiles. They also found that true-positive and false-positive identification rates decreased significantly as the number of drugs listed on the profile increased. They found that more years of pharmacy education seemed to improve the ability to detect drug interactions [28].

A study carried out by Ko et al. found that prescribers' knowledge of potential clinically significant DDIs is generally poor. The percentage of prescribers who correctly classified specific drug pairs ranged from 18.2% for warfarin and cimetidine to 81.2% for paracetamol (acetaminophen) with codeine and amoxicillin, with 42.7% of all combinations classified correctly. They found that for half of the drug pairs over one-third of the respondents answered 'not sure'; among those drug pairs, two were contraindicated [14].

A study conducted by Moges to assess physicians’ awareness of DDIs and common sources of information found that the physicians had low scores on DDI knowledge with an average of 33.3% correct responses. The percentage of physicians who correctly classified the drug pairs ranged from 12.9%, for the drug pair "praziquantel + rifampicin" (contraindicated drug combination) to 65.7%, for the drug pair "acetaminophen with codeine + amoxicillin" (non-interacting drug combination). This study disclosed that physicians who specialized in internal medicine other than cardiology had better DDI knowledge than those who reported having specialization in other areas such as dermatology/venereology, neurology, dentistry, and general surgery. The study also showed that pediatricians had better DDI knowledge than physicians who specialized in other areas. He found that those physicians who somewhat disagreed, somewhat agreed, or completely agreed with the statement "the risk of DDIs is high", and those who completely disagreed had lower DDI knowledge scores [20].

Selected Sources of Information on Drug Interactions include: Clinical Pharmacology (CD-ROM, Internet): complete database for drug interactions as well as clinically useful drug information; updated quarterly, Hansten and Horn's Drug Interactions Analysis and Management (manual): easy-to-use index that categorizes a drug interaction by clinical significance, along with a concise reference monograph discussing the interaction; updated quarterly, Handbook of Adverse Drug Interactions (manual);Adverse Drug Interactions Program (software, Internet): software searches for interactions between two and up to 25 drugs, Drug Interactions Analysis and Management (loose-leaf or bound manual); Drug Interaction Facts (loose-leaf or bound manual with software): information about drug–drug and drug–food interactions in a quick reference format, along with descriptive monographs of drug interactions selected on the basis of their potential to alter patient outcomes; updated quarterly [2].

A study carried out by Ko et al. [14] found that a quarter of the prescribers reported using personal digital assistants, and another quarter used printed material as a source to learn more about a potential DDI. The majority of the prescribers (68.4%) reported that they were usually informed by pharmacists about their patients’ potential exposure to DDIs. A study by Saverno et al. confirms the suboptimal performance of the pharmacy DDI software systems, in part, due to the failure of these systems to detect approximately one in seven clinically significant DDIs. The most poorly performing software system had a sensitivity of 0.23, meaning that approximately 77% of the DDIs evaluated would go undetected. Community pharmacies failed to detect approximately one in 12 clinically significant DDIs, while hospital pharmacy systems failed to detect approximately one in four DDIs. In addition, systems in other settings incorrectly categorized approximately one in seven of the DDIs evaluated. The study finds that additional efforts are needed to improve the ability of pharmacy software systems to detect clinically significant DDIs [25]. The data from this current study could also be useful to fill the existing information gap regarding this issue in Arabia Saudi and could serve as baseline data for other researchers.

The objectives of this study are to assess healthcare professionals' ability to recognize potential clinically significant DDIs and to examine the sources of information they use to identify potential DDIs and healthcare professionals' opinions on the usefulness of various DDI information sources in general hospitals of Buraydah in Kingdom of Saudi Arabia.

## Methods

### Sample size and method of sampling

The study was conducted in Buraydah, the regional capital of the Al-Qassim Region. The hospitals included in our study were: King Fahad Specialist Hospital, Buraydah Central Hospital, and Maternity and Children's Hospital. The total number of questionnaires distributed was 150 and the response rate was 90% which makes the sample size of this study 135 healthcare professionals. Healthcare professionals included are general practitioners, specialty doctors, pharmacists (B-Pharm), doctors of pharmacy (Pharm D), and nurses. They were selected by simple random sampling.

### Study design

This is a descriptive study. It is a questionnaire-based cross-sectional analysis. The survey was conducted among healthcare professionals in general service hospitals in Buraydah between November and December 2016. The self-administered questionnaire has been employed for data collection. This study aims to assess healthcare professionals` knowledge of DDIs and common sources of information in general hospitals of Buraydah. Inclusion criteria included licensed healthcare professionals working in general service hospitals in Buraydah. They included general practitioners, specialty doctors, pharmacists (B-Pharm), doctors of pharmacy (Pharm D), and nurses. Healthcare professionals have been selected by the sampling method and who were willing to participate in the study. Exclusion criteria as healthcare professionals working outside the general service hospitals in Buraydah, healthcare professionals who were not present during the time of data collection, and who did not voluntarily participate in the study.

### Operational definitions

Good DDI knowledge level: DDI knowledge test score with the mean (i.e., 5) and above out of 15.

Poor DDI knowledge level: DDI knowledge test score with the mean (i.e., < 5).

General practitioner: A medical doctor who has been registered and licensed by the Saudi Commission for Health Specialties (SCFHS) and whose practice consists of providing ongoing care covering a variety of medical problems in patients of all ages.

General hospital: A hospital providing ongoing care covering a variety of medical problems, such as internal medicine, gynecology, obstetrics, cardiology, etc. (includes general or specialized general hospitals).

Pharmacists: Those who completed a B-Pharm degree.

Pharmacy doctors: Those who completed a Pharm D degree.

None pharmacist clinicians: Healthcare professionals other than pharmacists (including health officers, nurses, etc.).

### Data collection instrument

The data collection tool was a structured questionnaire that was adapted from [20]. The healthcare professionals (general practitioners, specialty doctors, pharmacists (B-Pharm), doctors of pharmacy (Pharm.D), and nurses) of the three hospitals were provided with a copy of the questionnaire after an explanation of the study objectives. The healthcare professionals were provided with enough time to fill out the questionnaire.

The questionnaire consisted of three sections. The first section contained questions concerning healthcare professionals’ demographic characteristics that might account for differences in healthcare professionals’ ability to identify common DDIs and perceived opinions on the usefulness of usual DDI information sources. The second section contained questions set with four targets on healthcare professionals’ knowledge regarding potential DDIs of selected drug pairs. This segment contained 15 drug–drug pairs that are locally available and routinely prescribed. Only 15 drug pairs were selected for inclusion in this instrument assuming that more than this might decrease response rates. Among the 15 drug pairs, six are contraindicated; five could be used under monitoring and four had no known interaction. Without the aid of any reference, respondents were asked to classify each drug pair in one of four categories: (1) contraindicated; (2) may be used together but with monitoring; (3) no interaction, and (4) not sure. The "not sure" option was added to prevent guessing.

The last section contained questions regarding the sources that usually inform healthcare professionals about their patients’ exposure to potential DDIs along with five questions to explore their opinion on the usefulness of these information sources. Five closed-ended questions were used to assess the usefulness of DDI information sources, and the response option was the Likert scale (never, infrequently, sometimes, frequently, and always).

### Data entry and processing

The descriptive analysis was done using frequency distribution and percentage for demographic data and other responses to questions. Data were analyzed using Statistical Package for Social Sciences (SPSS) software (version 23).

### Ethical considerations

Ethical approval was obtained from the Regional Research Ethics Committee, Qassim province. A full explanation of the purpose of the study was made to the authorities of the respective hospital and the participants. Data collection was conducted after approval of the study by the medical and pharmacy directors of each hospital. All participants gave written informed consent before the start of the study. To assure confidentiality, participants were not asked to identify themselves by name.

## Results

### Socio-demographics of the participants

A total of 135 healthcare professionals were included in the study. The female respondents, 77 (57%) were more than the males 58 (43%). The ages of respondents were classified into four intervals, 89 (65.9%) of them reported in the first interval (from 21 to 30 years old), 29 (21.5%) in the second interval (from 31 to 40 years old), 14 (10.4%) in the third interval (from 41 to 50 years old), and the only 3 (2.2%) respondents in the fourth interval (more than 50). For the respondent's highest education level; 27 (20%) of them completed general practitioner, 16 (11.9%) of them completed specialty doctor, 21 (15.6%) of them completed pharmacy, 27 (20%) of them completed doctor of pharmacy, and 44 (32.6%) of them completed nursery. As for the departments, 13 (9.6%) of respondents were practicing in obstetrics and gynecology, 3 (2.2%) of them were practicing in I.C.U, 8 (5.9%) of them were practicing in surgery ward, 20 (14.8%) of them practiced in internal medicine other than cardiology, 9 (6.7%) of them were practicing in cardiology, 10 (7.4%) of them were practicing in Pediatrics, 17 (12.65) were practicing in Emergency department, 6 (4.4%) were practicing in OPD, 9 (6.7) were practicing in OR, 2 (1.5%0 were practicing in DPIC, and 38 (28.1%) were practicing in the pharmacy department as shown in Table 1.

**Table 1:** Socio-demographic characteristics of participants in general hospitals of Buraydah, 2016 (*N* = 135)

Variables	*n* [%]
Gender	
Male	58 [43.0]
Female	77 [57.0]
Age	
21–30	89 [65.9]
31–40	29 [21.5]
41–50	14 [10.4]
More than 50	3 [2.2]
Education level	
General practitioner	27 [20.0]
Specialty doctor	16 [11.9]
Pharmacist	21 [15.6]
Doctor of pharmacy	27 [20.0]
Nurse	44 [32.6]
Practice site	
King Fahad Specialist Hospital	59 [43.7]
Buraydah Central Hospital	39 [28.9]
Maternity and Children’s Hospital	37 [27.4]
Area of specialization	
Obstetrics and gynecology	
Intensive care unit(I.C.U.)	13 [9.6]
I.C.U	3 [2.2]
Surgery ward	8 [5.9]
Internal medicine other than cardiology	20 [14.8]
Cardiology	9 [6.7]
Pediatrics	10 [7.4]
Emergency department	17 [12.6]
Outpatient department (OPD)	6 [4.4]
Operating room (OR)	9 [6.7]
Drug and poison information center (DPIC)	2 [1.5]
Pharmacy	38 [28.1]
Years of professional experience	
< 1 year	30 [22.2]
1–5 years	50 [37.0]
6–10 years	31 [23.0]
11–15 years	10 [7.4]
16–20 years	11 [8.1]
More than 20 years	3 [2.2]

### Factors influencing healthcare professionals’ choice of new drugs

When asked what factor(s) was/were highly influencing their decisions on new drugs, 50 (37.6%) reported that only safety/including DDI influenced their drug selection the most. Of the majority of the HCPs, 55 (39.8%) reported that a combination of these factors influenced their drug selection the most. None of the respondents reported that patient requests for the drug or the cost influenced their drug selection. When asked specifically about the effect of a DDI`s risk on their decisions about a drug product, 124 (91.9%) of respondents agreed. Of those who agreed, 89 (65.9%) stated that it affected their decision very much as in Table 2.

**Table 2:** Factors influencing healthcare professionals` choice of new drugs in general hospitals of Buraydah

Variables	*N* [%]
Factors	
Safety/including drug interactions	50 [37.6]
Efficacy/effectiveness of the drug	11 [8.3]
Combination of safety and efficacy	19 [14.3]
Others*	55 [39.8]
Effect of DDIs` risk on decisions about a drug product	
Yes	124 [91.9]
No	11 [8.1]
Extent to which DDIs` risk affect decisions about a drug product	
No	11 [8.1]
A little	3 [2.3]
Somewhat	32 [23.7]
Very much	89 [65.9]

*Colleagues and/or specialists through referral and recommendations and combination of other factors

### Healthcare professionals’ history of encountering drug–drug interactions

Table 3 shows that more than half of the respondents, 78 (57.8%) indicated that they had never come across a patient who experienced a DDI that caused harm. Twenty-six (19.3%) of the respondents encountered DDIs several times that caused harm to the patient and 15 (11.1%) of the respondents came across DDIs that caused harm to the patients once in their practice.

**Table 3:** Healthcare professionals’ history of encountering DDIs in general hospitals of Buraydah

Item	*N* [%]
Had ever come across a drug–drug interaction that resulted in adverse outcomes	
Yes	57 [42.2]
No	78 [57.8]
Commonly observed adverse outcomes caused by drug–drug interactions	
No	78 [57.8]
Intoxication/over dosage	12 [8.9]
Hypotension	14 [10.4]
Bleeding	9 [6.7]
Therapeutic failure	11 [8.1]
Others*	11 [8.1]
Frequency of encountering a drug–drug interaction that caused harm	
No	78 [57.8]
Once	15 [11.1]
Twice	10 [7.4]
Three time	6 [4.4]
Several times	26 [19.3]

*Urticaria, persistent hypokalemia, tachycardia, angioedema, and a combination of other DDIs

### Healthcare professionals’ perceptions of drug safety and DDIs

**Fig. 1:**
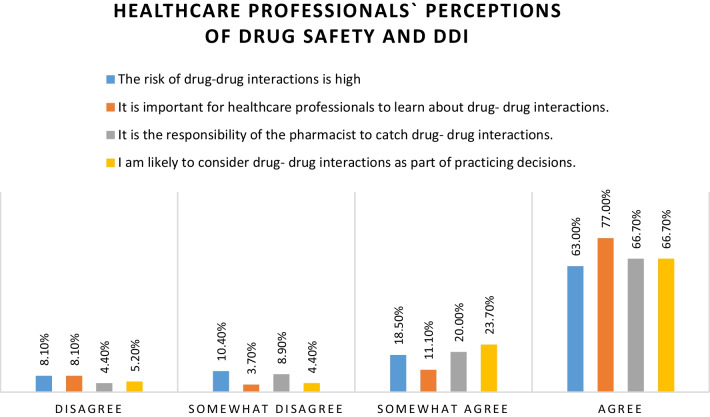
Healthcare professionals’ perceptions of drug safety and DDI

Over one-half of the study participants, 85 (63.0%) agreed that the risk for DDIs is high. Twenty-five (18.5%) of the respondents somewhat agreed that the risk for DDIs is high and 14 (10.4%) of the respondents somewhat disagreed with the presence of a higher risk of DDIs. The majority of respondents 104 (77.0%) agreed that it is important for prescribers to learn about DDIs. Fifteen (11.1%) somewhat agreed, whereas 11 (8.1%) disagreed about the importance of learning about DDIs (Fig. 1).

### Healthcare professionals’ usual information sources on drug–drug interactions

**Fig. 2:**
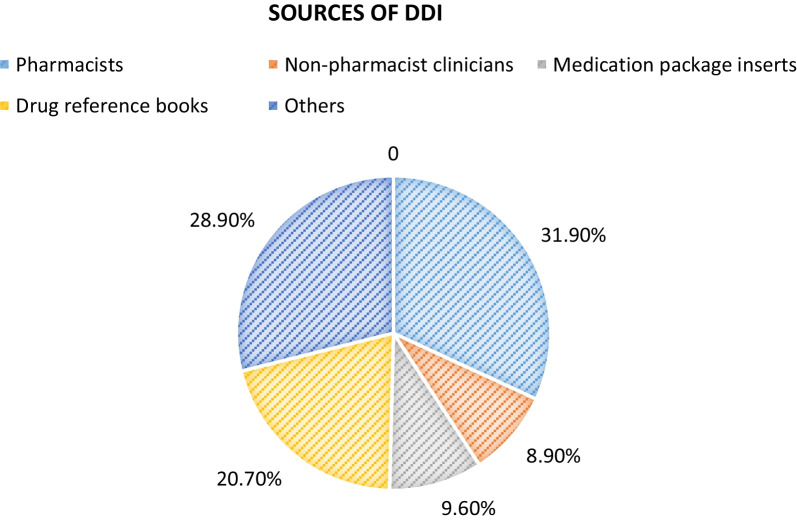
Healthcare professionals’ common information sources on DDIs in general hospitals of Buraydah

Pharmacists were the most cited information source used by 43 (31.9%) of the study participants followed by drug reference books which were used by 28 (20.7%) of the respondents. Thirty-nine (28.9%) of respondents were using other sources such as website references, databases, drug formularies, and a combination of other sources (Fig. 2).

### Healthcare professionals’ knowledge of potential drug–drug interactions

About half of the healthcare professionals in the present study had good scores (54.1%) on the DDI knowledge questions with an average of 5 correct responses. The percentage of HPCs that were correctly classified in the drug pairs ranged from 11.1%, for the drug pair "Allopurinol + Pyrazinamide" (contraindicated drug combination) to 66.7%, for the drug pair "acetaminophen with codeine + amoxicillin" (non-interacting drug combination). The average number of accurately categorized drug pairs was 5 with a standard deviation (SD) of 3.8. Pharmacists had the highest knowledge with an average of 7.95 and nurses had the lowest with an average of 2.70.

Nitroglycerin + sildenafil was correctly classified by over half of the respondents, 75 (55.6%) as contraindicated, and warfarin + fluconazole was correctly classified by over one-third, 47 (34.8%) of the respondents as contraindicated. Three of the six drug pairs that were considered as contraindicated were correctly classified by less than one-fourth of the respondents; warfarin + co-trimoxazole was correctly classified by 35 (25.9%) of respondents as a combination considered as contraindicated. Praziquantel + rifampicin by 34 (25.2%), and simvastatin + itraconazole (a combination considered as contraindicated) was correctly identified by 33 (24.4%) of respondents.

All of the five drug combinations to be prescribed with monitoring were correctly identified by about one-third of the HPCs; carbamazepine + clarithromycin by 42 (31.1%), digoxin + verapamil by 52 (38.5%), digoxin + clarithromycin by 43 (31.9%), atenolol + ranitidine by 58 (43.0%), and Carbamazepine + cimetidine by 38 (28.1%) of the HPCs.

Thirty-four (25.2%) of the respondents chose the response category "not sure" for the drug pair "acetaminophen with codeine + amoxicillin" and 76 (56.3%) chose the response category "not sure" for the drug pair "Allopurinol + Pyrazinamide". About one-half of the respondents answered "not sure" for three of the drug pairs that were contraindicated (allopurinol + pyrazinamide, praziquantel + rifampicin, and warfarin + co-trimoxazole); for two of the drug pairs which had no any interaction (digoxin + sildenafil and metformin + erythromycin) and about one-third for the two of the drug pairs which were to be used with monitoring (carbamazepine + digoxin and clarithromycin) (Table 4).

**Table 4:** Healthcare professionals` knowledge of potential drug–drug interactions in general hospitals of Buraydah

Drug pairs	Contra-indicated *N* [%]	Could be used under monitoring *N* [%]	No interaction *N* [%]	Not sure *N* [%]
Acetaminophen with codeine + amoxicillin	6 [4.4]	5 [3.7]	90 [66.7]*	34 [25.2]
Carbamazepine + clarithromycin	26 [19.3]	42 [31.1]*	16 [11.9]	51 [37.8]
Digoxin + verapamil	24 [17.8]	52 [38.5]*	10 [7.4]	49 [36.3]
Digoxin + clarithromycin	13 [9.6]	43 [31.9]*	22 [16.3]	57 [42.2]
Digoxin + sildenafil	13 [9.6]	24 [17.8]	40 [29.6]*	58 [43.0]
Metformin + erythromycin	2 [1.5]	13 [9.6]	58 [43.0]*	62 [45.9]
Nitroglycerin + sildenafil	75 [55.6]*	15 [11.1]	5 [3.7]	40 [29.6]
Simvastatin + itraconazole	33 [24.4]*	26 [19.3]	15 [11.1]	61 [45.2]
Warfarin + cimetidine	26 [19.3]	38 [28.1]*	18 [13.3]	53 [39.3]
Atenolol + ranitidine	1 [0.7]	12 [8.9]	58 [43.0]*	64 [47.4]
Carbamazepine + cimetidine	14 [10.4]	38 [28.1]*	22 [16.3]	61 [45.2]
Warfarin + fluconazole	47 [34.8]*	24 [17.8]	11 [8.1]	53 [39.3]
Allopurinol + pyrazinamide	15 [11.1]*	12 [8.9]	32 [23.7]	76 [56.3]
Praziquantel + rifampicin	34 [25.2]*	15 [11.1]	10 [7.4]	76 [56.3]
Warfarin + co-trimoxazole	35 [25.9]*	20 [14.8]	16 [11.9]	64 [47.4]

*Correct responses

### Healthcare professionals’ perceived usefulness of DDIs information sources

Table 5 shows that for the statement regarding how often the DDI information changes their initial prescribing decisions, 49 (36.3%) respondents reported the information always changed their initial prescribing decisions, and only 8 (5.9%) of respondents chose the response category "never" for this statement. The DDI information provided by their DDI information sources was always new to 40 (29.6%) of the HPCs. Fifty-seven (42.2%) of the respondents reported that the information was always relevant to the patient. The DDI information was always sufficient to manage the interaction for 49 (36.3%) of the respondents. Concerning the future usefulness of the information, about three-quarters 103 (76.3%) of the study participants reported that the information is always useful in the future.

**Table 5:** Healthcare professionals’ perceived usefulness of DDIs information sources in general hospitals of Buraydah

Questions	Never *n* [%]	In infrequently *n* [%]	Sometimes *n* [%]	Frequently *n* [%]	Always *n* [%]
How often does the drug interaction information change your initial practicing decisions?	8 [5.9]	15 [11.1]	36 [26.7]	27 [20.0]	49 [36.3]
How often is the drug interaction information new to you?	8 [5.9]	20 [14.8]	37 [27.4]	30 [22.2]	40 [29.6]
How often is the drug interaction information relevant to the patient?	13 [9.6]	18 [13.3]	34 [25.2]	13 [9.6]	57 [42.2]
Is the drug interaction information sufficient for you to manage the interaction?	10 [7.4]	19 [14.1]	30 [22.2]	27 [20.0]	49 [36.3]
How often is the drug interaction information useful to you in your future practicing?	4 [3.0]	3 [2.2]	11 [8.1]	14 [10.4]	103 [76.3]

## Discussion

This study assessed healthcare professionals’ ability to recognize potential clinically significant DDIs and examined the sources of information they use to identify potential DDIs in general hospitals of Buraydah in the Kingdom of Saudi Arabia. To attain this, the study identified healthcare professionals` socio-demographic characteristics, and common methods of DDI information sources and tested healthcare professionals' knowledge of DDIs.

Studies on HCPs' knowledge regarding drug–drug interactions are limited. To our knowledge, this is the first study done in Saudi Arabia to evaluate the HCPs' knowledge of drug–drug interactions.

In our study, the female respondents 77 (57%) were more than the males. Among these, there were 16 general practitioners (11.9%), 21 pharmacists (15.6%), and 44 nurses (32.6%). A study carried out by Al-Arifi et al. [1] had 24 physicians (26.7%), 31 pharmacists (34.4%), and 35 nurses (38.7%). In the same study, more than 70% of physicians and pharmacists were male. In our study 50 (37.0%) of respondents had been in their practice for 1–5 years compared to a study conducted by Moges [20] which reported that 65 (46.4%) had been in their practice for less than 10 years.

Healthcare professionals need to be aware of the preventive ways of drug–drug interactions and learn about their importance to be able to control their effects, and hence get the intended benefits of the treatment.

In our study, we found that 50 (37.6%) reported that only safety /including DDI influenced their drug selection the most, but none of the respondents reported that patient requests for the drug or the cost influenced their drug selection. In a study conducted by Buusman et al. [6] indicates that four different types of factors influence the general practitioners’ (GPs) choice of the drug: the price, internal, and external factors, and a complex system of factors related to the actual consultation. Another study conducted by Cossens et al. [7] found that the majority of hospital doctors (HDs) ranked efficacy as the most important drug characteristic, whereas general practitioners were generally more concerned with side effects. They also found that fundholding GPs ranked cost more highly than their non-fundholding counterparts and GPs working in single-handed practices were more influenced by specialists and company representatives than were GPs working in groups.

The consequences of being exposed to an interaction are not easy to be overlooked. Juurlink et al. [12] reported that the risk of hospitalization substantially increases for those patients exposed to a DDI. However, in our study, we found that the majority of the respondents, 78 (57.8%) indicated that they had never come across a patient who experienced a DDI that caused harm.

Healthcare professionals need to be aware of the risks of DDIs to enhance drug safety and ensure proper management of DDIs. In this study, we found that 85 (63.0%) study participants agreed that the risk for DDIs is high. This finding is promising, because if they perceive the risk of DDIs as high, then they will be more careful when dealing with drugs and will work harder to identify and prevent them.

In this study, pharmacists were the most cited information source used by 43 (31.9%) of the study participants, this corresponds with a study in the USA by Malone et al. [17] which revealed that the majority of the physicians 650(68.4%) reported that they consulted pharmacists about DDIs and in a study carried by Ko et al. [14] which found that the majority of the prescribers (68.4%) reported that they were usually informed by pharmacists about their patients' potential exposure to DDIs.

DDIs are one important and unrecognized type of medication error that predisposes patients to hospitalization and increases the cost burden on healthcare systems [13]. Physicians, nurse practitioners, and pharmacists constitute the group of providers in closest proximity to patients receiving medications. Thus, understanding the degree to which these providers can recognize an interaction and identify a proper management strategy is vital to developing new methods to reduce DDIs [5]. In the present study, half of the healthcare professionals (54.1%) had a good score on the DDI knowledge questions with an average of 5 correct responses. This is about higher than the average reported by Moges [20] 33.3%, and higher than the studies conducted in the USA by Glassman et al. [9] and Ko [15], which reported correct responses of 44% and 42.7%, respectively. The higher score in this study might be because of variations in sample size. A study of prescribers' knowledge of interactions by Glassman et al. [9] found that clinicians correctly classified drug–drug combinations as interacting or not about 50% of the time. Another study conducted by John [11] found that only 31 (36.0%) of 86 clinically significant drug interactions (CSDIs) were correctly identified by physicians. They stated that although it could be argued that CSDIs were appropriately managed through clinical vigilance and laboratory monitoring, the surprisingly low rate of physician awareness (only 36% of all CSDIs were correctly identified) suggests otherwise. In the same study, they found that poor physician recognition of CSDIs is not confined to HIV treatment and is also seen with other commonly prescribed medications, such as warfarin.

In our study, we found that the percentage of HPCs who correctly classified the drug pairs ranged from 11.1%, for the drug pair "Allopurinol + Pyrazinamide" (contraindicated drug combination) to 66.7%, for the drug pair "acetaminophen with codeine + amoxicillin" (non-interacting drug combination). A study carried out by Ko et al. [14] found that the percentage of prescribers who correctly classified specific drug pairs ranged from 18.2% for warfarin and cimetidine to 81.2% for paracetamol (acetaminophen) with codeine and amoxicillin. Another study conducted by Moges [20] found that the percentage of physicians who correctly classified the drug pairs ranged from 12.9%, for the drug pair "praziquantel + rifampicin" (contraindicated drug combination) to 65.7%, for the drug pair "acetaminophen with codeine + amoxicillin" (non-interacting drug combination). The findings in our study might be because we included all healthcare professionals in the study while in their studies they included only the physicians, this means that in our study there were variations in the knowledge due to variations in the level of education.

In our study, we found that warfarin + fluconazole was correctly classified by over one-third, 34.8% of the respondents as contraindicated. A study carried out in Saudi Arabia by Al-Arifi et al. [1] to assess HCPs’ knowledge of warfarin–drug/herb interactions revealed that warfarin + fluconazole has a recognition rate of 47.8%. This could increase the risk of bleeding if not immediately recognized and corrected.

In our study, we found that about one-half of the respondents answered "not sure" for three of the drug pairs that were contraindicated (allopurinol + pyrazinamide, praziquantel + rifampicin, and warfarin + co-trimoxazole). A study by Ko et al. [14] found that for half of the drug pairs over one-third of the respondents answered 'not sure',among those drug pairs, two were contraindicated. Moges [20] reported that over one-third of the respondents answered "not sure" for three of the drug pairs that were contraindicated. A study by Glassman et al. [9] found that 28% of clinicians were not sure whether sildenafil and isosorbide interacted, 27% were uncertain whether there was an interaction between cisapride and erythromycin, and 43% were not sure whether concomitant use of phenelzine and sertraline presented problems, all of these interactions are potentially life-threatening.

While not all ADEs are predictable, exposure to a clinically significant DDI is a preventable medical mistake [24]. Therefore, hospital managers should investigate ways to prevent drugs with potentially dangerous DDIs from reaching the patient. One way of realizing this goal is to identify factors that encourage or discourage healthcare professionals’ DDI knowledge.

## Conclusions

The present study revealed that healthcare professional's DDI knowledge was inadequate. Level of education was significantly associated with healthcare professionals’ DDI knowledge. The drug pair "acetaminophen with codeine + amoxicillin" was the most correctly identified among the other pairs by healthcare professionals. A better DDI knowledge level was seen with pharmacists. In this study, pharmacists were the most cited source of information by healthcare professionals. Healthcare professionals should update their DDI knowledge through continuing education and should improve their familiarity with DDI information sources. These updated educations help to provide the appropriate therapeutic outcomes. Furthermore, this survey can serve as a preliminary study and help understand the knowledge of HCPs on drug–drug interactions in Saudi Arabia.

Limitations: Healthcare professionals in government hospitals were too busy, which might affect the representativeness of the sample drawn from governmental hospitals.

Recommendations: Based on the findings of this study the following recommendations are forwarded: for healthcare professionals should update their DDI knowledge through continuing education and should improve their familiarity with information sources such as smartphone applications, compendia of drug products (such as American Hospital Formulary Service—AHFS-Drug Information, Martindale, and Physician’s Desk Reference) that assist in identifying potential DDIs.

## Data Availability

It is applicable.

## Abbreviations

ADEs: Adverse drug effects
AHFS: American Hospital Formulary Service
CSDIs: Clinically significant drug interactions
DDI: Drug–drug interactions
GPs: General practitioners
HCPs: Healthcare professionals
OR: Odd ratio
SD: Standard deviation
SPSS: Statistical Package for Social Sciences
WHO: World Health Organization
